# The Purpose of Time-Motion Studies (TMSs) in Healthcare: A Literature Review

**DOI:** 10.7759/cureus.29869

**Published:** 2022-10-03

**Authors:** Poonam S Kalne, Ashok M Mehendale

**Affiliations:** 1 School of Epidemiology and Public Health, Datta Meghe Institute of Medical Sciences, Wardha, IND; 2 Preventive Medicine, Datta Meghe Institute of Medical Sciences, Wardha, IND

**Keywords:** efficiency, healthcare, time and motion studies, standardization, methods

## Abstract

By utilizing time study methodologies, one can ascertain how long it takes a skilled person to complete a task with a specific level of quality. Time study aids in the selection of different job execution options as well as in the determination of the workforce required for a certain task, and thus assist in increasing manpower efficiency. It also aids in the acquisition of plants and machinery. By elaborating on the definition of "time-motion studies" (TMSs) as it is used in the biomedical literature and presenting justification based on full knowledge of that definition, this work aims to contribute to the standardization of TMSs. In TMSs, which is a type of quantitative data gathering, an outside observer records the motions and time necessary to complete an activity, together with an analysis aimed at increasing productivity. This paper suggests that, according to the researchers, the term is used to designate a range of investigations, the gathering and/or analysis of the length of one or more occurrences. A detailed analysis of all the available literature is done in this paper to get knowledge about TMS and its use in healthcare. Also, a comprehensive overview of many methodologies applied in works that are classed or referred to as TMSs is provided in this paper. Time motion investigations were first introduced in industrial engineering at the beginning of the twentieth century. Since then, they have been extensively employed by biological researchers, and because there is currently interest in parts of clinical workflow, they have attracted attention. However, combining the findings from different studies has proven challenging because there is a lot of variation in how techniques are used and reported. Although efforts have been made to uniformly publish these data and outcomes, there is still confusion about what TMSs are. A shared understanding of time and motion (TAM) research, as well as a proper acknowledgment of the various approaches it comprises, is a critical step toward standardization and validation. In this review paper literature analysis is done to discover what is known as TMSs to achieve the mentioned purpose.

## Introduction and background

A systematic understanding of the link between time and motion (TAM) is provided through method study and work study. While motion study is used to enhance labor methods, time study's primary goal is to set a standard time. The bond between the two is long-standing. Resource management techniques like time study and motion study can lead to increased output and performance. The systematic documentation and critical evaluation of the proposed and actual working procedures is referred to as a "method study." It aids in the application of simpler and more effective strategies for lowering construction costs and speeding up the process. It also improves workers' working conditions and the environment by preventing unnecessary manpower movement [1]. There is a strong need to discuss and apply time-motion studies (TMSs) to improve the working efficiency in healthcare [2]. By considering this need an attempt is made to collect the available data on TMSs by searching different research papers through different sources.

Because of increasing concern about inefficiencies and waste of material resources, the study of industrial processes gained a lot of interest in the early twentieth century [3]. This paper discusses TMSs and their purpose in healthcare in detail, so that it will become useful to the health professional and researchers in future research work [4,5]. Frederick Taylor (1856-1915) committed his life to know more about the time study method, believing that it was the most important subject he had ever worked on. With this time study method for speeding up processes, he made a contribution to the developing field of "scientific management." Time studies were conducted using extensive stopwatch observations of people performing a range of occupations at their most fundamental levels (for instance, how long it takes the shovel to swing back before the load is thrown a particular horizontal distance and height). This another concept of "motion study" was created by Frank and Lilian Gilbreth, two of Taylor's pupils. The motion study method aims to improve a procedure's effectiveness by minimizing the number of moves required to finish it. Following the combination of these two methodologies, TMS, a well-known scientific management tool, was created. The American Society of Mechanical Engineers devised Taylor's time study method in the beginning to show how the same concepts might be used to a variety of human activities. The Gilbreth launched their business in 1914 examining inefficiencies in the healthcare and life sciences industries, using their motion studies approach [6]. TMS has since gained widespread acceptance among hospital executives and academics who have utilized it to investigate healthcare costs and inefficiencies before quickly shifting their focus to patient safety and quality is paramount. Informatics and information technology systems have recently become more common in the fields of healthcare and the life sciences, which has raised the need to examine and assess these systems because they may have a big impact on the effectiveness, cost, and quality of healthcare [7-13]. One of the most frequently cited obstacles to the successful adoption of electronic health records is the increasing amount of paperwork. Measuring documentation and other workflows are more effective when they are finished on time-related issues, which has become a popular study goal. Time motion studies play a crucial role in satisfying this goal [1,14].

There is sincere interest in merging TMS data to shed light on the workflow, inefficiencies, patient safety, and quality of healthcare as well as, more recently, to assist in the purchase and deployment of health information technology [11,12]. In order to analyze the workflow and working patterns in the healthcare industry as well as in other disciplines, this review paper on time motion study will unquestionably supply the necessary knowledge. When the findings of researches were combined, it was found that it is difficult to compare the conclusions because of the significant discrepancies in the design, implementation, and data reporting of various TMSs. The inconsistency of the activity categorizations and the absence of methodological standardization make it challenging to combine the findings from different TMSs. The goals of this review paper are to examine a representative sample of biomedical literature categorized as TMSs to ascertain the method's current scope, thoroughly describe the various methodologies used in those papers, present a TMSs disambiguation schema, and use TMSs to improve operating efficiency, productivity, tool time, and support time while lowering idle time and also to boost productivity with the help of time motion studies by assuring the most efficient use of human resources, machines, and materials, as well as to produce the highest quality product feasible [1,3,11].

Methodology

The MeSH (Medical Subject Headings) words for the years 2011 to 2022 were used to search the PubMed database. Our definition focused on the phrase "Time Motion Studies" as it is used in biological literature right now, especially in the healthcare sector. In the title or abstract of empirical studies that were published in English, the terms "time motion study" or "time and motion study" were studied. Due to the fact that the PubMed search engine reads dashes as spaces, the inclusion of "time-motion research" had no effect on the results.

Using either title or abstract, this search method was created to locate articles in which the author made it obvious that a TAM research had been conducted. We chose the MeSH major topic "Time and Motion Studies" to maintain evaluation effectiveness while extending its scope. Also we examined the terms "time motion studies," "time motion studies as defined by MeSH," and "time motion studies as seen by academics." In this paper we evaluated a convenience sample of articles published in the last ten years that either listed "Time and Motion Studies [MeSH]" as one of their main topics or that have the phrase cited in the body text or abstract. In this paper, we had omitted editorials, comments, and reviews from the search as we were only interested in journal papers because time motion research procedures in empirical studies were of interest to us. Using this search strategy, certain papers were examined, where the author had explicitly mentioned that time motion research had been done, either in the title or the abstract. Additionally, the main area of research was evaluated and categorized. It also included what MeSH refers to as "time motion studies" according to specialists.

("Time and Motion Studies"[Majr] OR "time and motion study"[Title/Abstract] OR "time motion study"[Title/Abstract]) AND "Health Care Category"[Mesh] AND English[lang] AND "2011/01/01"[PDAT] : "2022/01/01"[PDAT] AND Journal Article[ptyp] AND Observation Study[pytp] NOT Editorial[ptyp] NOT Review[ptyp] NOT Comment[ptyp] NOT Letter[ptyp] NOT Systematic review[ptyp] NOT Meta-analysis[ptyp] NOT Clinical Study[ptyp] was the final search strategy.

## Review

**Figure 1:**
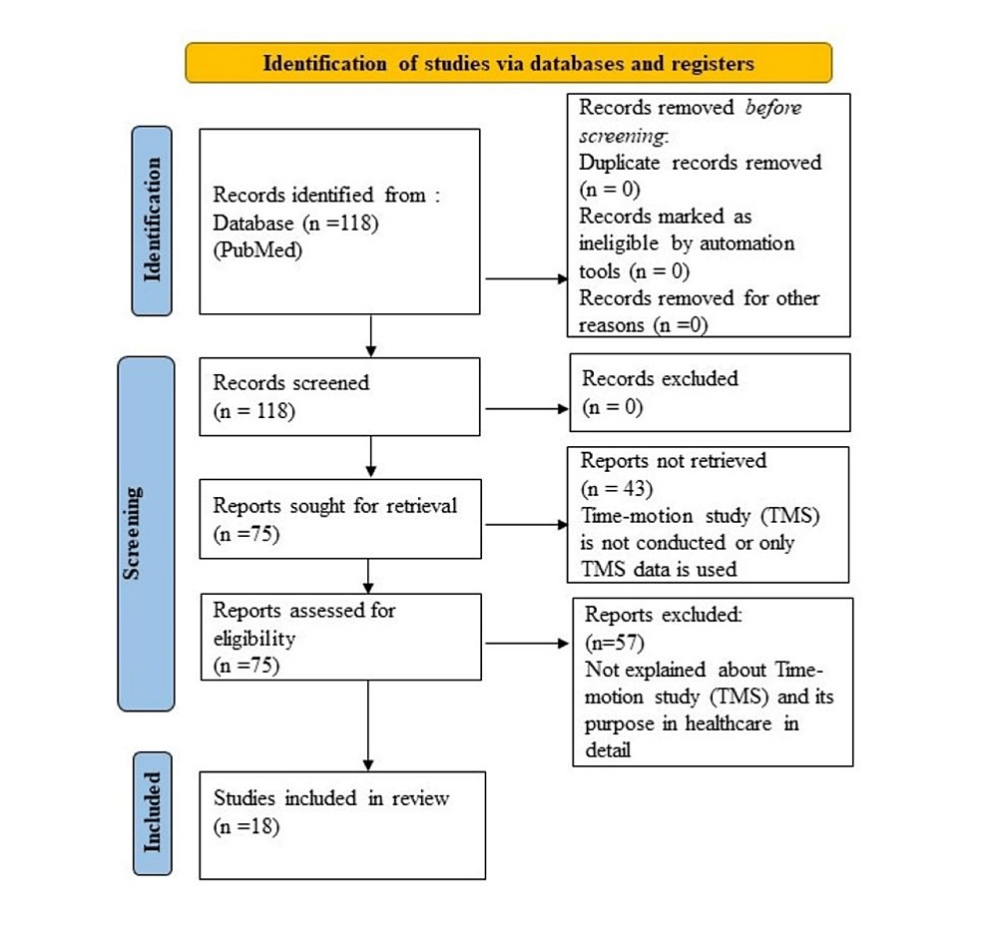
PRISMA flow diagram to show the selection of eligible articles TMS: Time-motion study; PRISMA: Preferred Reporting Items for Systematic Reviews and Meta-Analyses

The query was ran from 2011 to 2022 and retrieved in to 118 citations. No additional exclusion criteria was applied; our goal was to evaluate any empirical work that was either categorized as TMS by the authors or by MeSH. Out of 118 studies, 43 articles were excluded as they did not fit the selection criteria (not a TMS or only TAM data was used), leaving 75 articles for full assessment. The assessment was carried out on the entire article, with very few exclusions and only when there was no question regarding the approach being stated. Out of those full text 75 articles, 57 articles that did not explain TMS in detail were not included in the assessment. Thus, total 18 articles were used for the final study. Figure 1 represents a Preferred Reporting Items for Systematic Reviews and Meta-Analyses (PRISMA) flow diagram to show the selection of eligible articles. 

 

TMSs and their utilization in healthcare

The TAM technique was developed at the start of twentieth century by Frederick Taylor and Frank, and Lilian Gilbreth to improve the efficiency of industrial processes. The method was first applied by the Gilbreths in 1914 to investigate task times in healthcare facilities [15]. This traditional review provides a detailed description of the various methodologies used in different papers and examines a representative of the biomedical literature that is mentioned in or categorized as "TAM investigations" to ascertain the method's current application [16-19]. According to the current review, a TMS is a method used in business, healthcare, and other fields to assess the productivity of employees. This approach divided a difficult task into manageable steps and recorded the time it took for each action. The actions an employee took to complete each task were then evaluated in order to analyze the results. By determining the exact time taken for each movement, the paper suggests that TMS can be used to identify and eliminate unnecessary motion [8,9,11-13].

This review emphasizes the cost-saving potential of TMSs. With the help of TMS, one can regulate expenses, enhance working conditions, inspire staff, and boost productivity by developing a "standard" job technique. It should be emphasized that TMSs are typically only applicable for repetitive jobs, therefore approaches may vary by occupation. To observe pharmacists in multiple practice settings and record their activities to gauge inefficiencies, the daily activities of pharmacists in academic, retail, clinical, hospital, ambulatory, and clinical contexts were observed using TAM approaches. Pharmacists have a wide range of duties and work in many different environments. However, pharmacists are not available at low cost and need to be employed efficiently. One research revealed that a pharmacist's day is largely devoted to the creation of prescriptions, interactions with clinicians, and analysis of medical records. Additionally, it was assumed that roughly half of their time was spent on unnecessary work [20].

A TMS was carried out in June and July 2010 with seven first-year, second-year, and third-year residents working on the general medicine service at the New York-Presbyterian/Columbia University Medical Center to comprehend the purposes for which inhabitants used computers. Time they spend by using computers is compared to other activities. During a single day shift, a skilled observer recorded every activity using an iPad app and collected field notes. The activities were recorded using a proven clinical activity taxonomy that had been modified to more thoroughly define computer-based activities. According to the study, residents interacted with computers for over 50% of their shift time, as opposed to less than 10% of their shift time, when they were in direct touch with patients [21].

A TMS is a technique that counts the seconds taken by a subject to complete each job in order to figure out the workflow, ensures effectiveness, and simplifies processes. In hospitals and clinics, time motion studies are frequently used to evaluate the efficiency and time management of pharmacists, doctors, and nurses [22]. In one study, patient visits were analyzed by a researcher with expertise in TMS methodologies. Due to the lack of clearly defined phases of observed service, the study technique combined a work-sampling inquiry with a TAM analysis. This experiment was carried out using direct observation and timing methods that are typically utilized in TMSs. The project received approval from the Pacific University Institutional Review Board [23]. This TMS study compared how stockroom managers interacted with supply networks at Senegal's service delivery locations. This study included three objectives: to understand how employees at service delivery sites use their time when working on the family planning (FP) supply chain; to determine the applicability of self-administered timesheets (STs) versus continuous observations (COs) as the gold standard for measuring staff members' time in the Senegal’s setting; and to describe the research team about the expenses involved in data gathering for both STs and COs [24].

An extensive qualitative investigation combined with a direct observational TMS made it possible to evaluate work patterns and time usage, as well as problems with work planning and time management, and also gave number of systemic, community-based, and personnel factors that affect health workers performance in their jobs. The results of one study demonstrated that how employees use their time and how it differed in tribal and non-tribal location to boost worker productivity. Researchers and managers might design suitable tasks and routinely assess work habits. The paper suggested that TAM techniques were had been used successfully to describe a normal doctor workday and to offer suggestions for streamlining procedures for a long time. The TAM technique was utilized in numerous other studies to evaluate the efficiency of various healthcare professional cadres and encourage the usage of standardized practices. This study was exceptional because it examined supervisors' perceptions of the efficient operation of front-line health workers (FLHWs) and had a significantly larger sample size than is typical for a TMS. Additionally, it analyzed the relationships between different cadres' job duties, tracks geographical variance, and records complex work patterns [25].

In a study of TAM, an observer counts and notes each action a healthcare professional takes, as well as the precise duration of each action. As a result, it can accurately capture the length, frequency, and timing of an activity as well as the order of events. Recently, TMSs were utilized to gather quantitative data on workflow and work patterns in a variety of healthcare settings, including community pharmacies outside of Australia and hospital pharmacies in Australia. This study showed that With software installed on a tablet computer, such data can be simply captured electronically [26].

The degree and pattern of health extension workers' (HEWs') activity was clarified by TAM investigation in another study. Some studies examined how health workers use their workdays globally. But this study was challenging, expensive, and prone to measurement and sample errors and it provided insightful data on the time management practices of Ethiopia's HEWs while addressing some of the flaws in earlier studies [27]. Using a TAM analysis and a comparison methodology, this study emphasized the differences in the meaning of administrative labor in various hospital settings as well as the factors that accounted for these findings. The focus of this alternate perspective on nurses' administrative work was how it was related to the scope and structure of nursing practices. According to an idealized patient-centered model of nursing, the majority of research either complained about administrative work and its burdens or criticized it by pointing out how it affected the amount of time spent at the bedside with patients. The purpose of this study was to go beyond this framework and show that organizational factors, not time spent, were the primary determinant of nurses' perceptions of documentation and organizational activities (DOA) and its burdens [28].

Another study comparing the duration of manual measurements with 3D scanning was based on ongoing monitoring of significant events. The authors devised and assessed the study procedure and apparatus using Suggested Time and Motion Procedures (STAMP) developed by Zheng, Guo, and Hanauer (2011). All measuring tasks were created by the principal investigator and one anthropometrist from Body Imaging for Nutritional Assessment Study (BINA), and they also created start/stop time signals based on those activities. To increase accuracy, the time was recorded by a single observer using a stopwatch [29].

One research provided insightful information on how nursing staff members spend their time working and giving nursing care in a residential aged care (RAC) home. The three tasks that took the greatest time and were done the most frequently in nursing were administering medications, documenting, and verbal communication. Mostly activity categories had an average duration of less than one minute [30]. The labor activities performed by ICU nurses were described using an observational TAM research in another paper. This study's observational methodology was chosen because it enabled the recording of all activities taking place over a predetermined period of time in a natural setting. This TAM approach gave detailed information about how much time a participant needs to complete activities. This approach often uses a small sample size, but it has the capacity to gather a lot of data [31].

Nursing managers can use the findings of the TMS to evaluate staff performance and project costs. The data may also be utilized to create senior care programs that work as well as provide prospective standards for studies on nursing homes. It also supported the government's financial allocation by precisely determining the amount of time necessary to carry out each sort of care activity in order to meet a resident's critical care requirements. It is necessary to conduct more study to ascertain how oral communication helps other care modalities and how indirect care activities promote direct care [32].

When providing healthcare to patients, all pertinent activities should be identified and appropriate times for each activity should be measured. TAM research design is very helpful in satisfying this purpose. This knowledge might help with patient management and boost the effectiveness of the healthcare system. Because TAM research requires a lot of work, it frequently has limited participant numbers. While following the subject for a predetermined period of time, the observer meticulously records the time and the activities they are participating in. The work of participants is typically divided into its component sections followed by recording the overall amount of time spent on each category [33,34].

There were several benefits to the TMS that were discussed in one paper [1]. It first added to the information gathered during the lay health workers' (LHWs') interviews. The information made clear how context affected the programs which were carried out. Numerous novel practices were discovered as a result of the observations that might not have been known otherwise. This sub-study gave us unexpected chances to have casual, unplanned interactions with the team leaders and LHWs. The knowledge we gained from these exchanges has substantially expanded our understanding of the day-to-day realities of LHWs' work. These insights revealed opportunities to investigate fresh issues due to client engagement [35,36].

Limitations

Utilizing just one database (PubMed) instead of additional biomedical bibliographic databases or business/management literature could have constrained the variety of papers found. Additionally, these results may not be applicable to other domains (like industrial engineering) because they are restricted to TAM research in the biological industry, notably in healthcare. Also while sorting full text articles, the studies which only used TMS data and didn’t elaborate TMSs were excluded. Table 1 represents relevant literature used to review the current research paper. 

**Table 1:** Relevant literature used to review the current research paper TMS: Time-motion study

Search	Title	Authors	Year	Study type
1.	What does a pharmacist do? A time and motion study	Negaard B et al. [20]	2020	Observational TMS
2.	A time motion study of community mental health workers in rural India	Chebolu- Subramanian V et al. [8]	2019	Observational TMS
3.	How long do nursing staff take to measure and record patients' vital signs observations in hospital? A time-and-motion study	Dall'Ora C et al. [2]	2021	Observational TMS
4.	Time and motion study of pharmacist prescribing of oral hormonal contraceptives in Oregon community pharmacies	Frost TP et al. [23]	2019	Observational TMS
5.	Comparing time and motion methods to study personnel time in the context of a family planning supply chain intervention in Senegal	McElwee E et al. [24]	2018	Observational study
6.	Time motion study using mixed methods to assess service delivery by frontline health workers from South India: methods	Singh S et al. [22]	2018	Mixed-method study
7.	Work pattern of neurology nurses in a Chinese hospital: A time and motion study	Yu P et al. [25]	2019	Observational TMS
8.	Considering pharmacy workflow in the context of Australian community pharmacy: A pilot time and motion study [22]	Cavaye D et al. [26]	2018	Pilot observational TMS
9.	Ethiopia's health extension workers use of work time on duty: time and motion study	Tilahun H et al. [27]	2017	Observational TMS
10.	The content and meaning of administrative work: a qualitative study of nursing practices	Michel L et al. [28]	2017	Comparative case study
11.	A collaborative, mixed-methods evaluation of a low-cost, handheld 3D imaging system for child anthropometry	Conkle J et al. [29]	2019	Mixed-method study
12.	The work pattern of personal care workers in two Australian nursing homes: a time-motion study	Qian S et al. [34]	2012	Observational TMS
13.	The potential of task-shifting in scaling up services for prevention of mother-to-child transmission of HIV: a time and motion study in Dar es Salaam, Tanzania	Naburi H et al. [15]	2017	Observational TMS
14.	Multiple and mixed methods in formative evaluation: Is more better? Reflections from a South African study	Odendaal W et al. [36]	2016	Mixed-method study
15.	The provision of TB and HIV/AIDS treatment support by lay health workers in South Africa: a time-and-motion study	Odendaal WA et al. [35]	2014	Observational TMS
16.	Time and labor costs associated with administration of intravenous bisphosphonates for breast or prostate cancer patients with metastatic bone disease: a time and motion study	Xie F et al. [33]	2014	Observational TMS
17.	Understanding the work of intensive care nurses: a time and motion study	Abbey M et al. [31]	2012	Descriptive quantitative structured observational study
18.	Nursing staff work patterns in a residential aged care home: a time-motion study	Qian S et al. [30]	2016	Observational TMS

## Conclusions

Our study shows that there is a strong need to clearly specify TAM investigations, their definition, and their scope. One of the factors of interest in a lot of studies now being defined by this phrase is the duration of an event. The employment of an outside observer who continuously records time data is expressly mentioned. To make it easier for TMS researchers and reviewers to comprehend the distinctions between the various treatments, we have provided an overview of each technique covered by the current worldwide definition of TMS. The primary objective of the anticipated actions in this series is to standardize TAM research. With the aid of this review's assistance in providing a comprehensive account of the method used, it will be feasible to conduct further research into the differences between recording multi-tasking, defining interruptions, educating observers, a single clinical TMS task ontology will be developed, along with an evaluation of inter-observer reliability.

This paper is also useful to do research work in other fields along with healthcare on TMS. The literature review will definitely add some important knowledge regarding TMSs, which will eventually become helpful to the researchers and medical professionals to utilize and apply this knowledge in understanding the workflow and working pattern of staff members and, thus, to improve the working efficiency of healthcare workers.
